# Transition tips: How can we be better leaders as family physicians?

**DOI:** 10.4102/safp.v67i1.6055

**Published:** 2025-02-25

**Authors:** Chantelle C. van der Bijl, Arun Nair, Klaus B. von Pressentin

**Affiliations:** 1 Department of Family Medicine, Faculty of Health Sciences, University of the Free State, Bloemfontein, South Africa; 2 Department of Family Medicine, Robert Mangaliso Sobukwe Hospital, Kimberley, South Africa; 3 Department of Family, Community and Emergency Care, Faculty of Health Sciences, University of Cape Town, Cape Town, South Africa

Good leadership is a crucial need in our evolving South African healthcare system, and newly qualified family physicians have a key role to play as leaders in primary care teams. Leadership is commonly associated with positions of authority and decision-making related to management processes, but it encompasses much more. The journey from being a registrar to a medical specialist can be filled with challenges and growth opportunities for newly qualified family physicians. The Next5 is a special interest group of the South African Academy of Family Physicians (SAAFP) that supports newly qualified family physicians in their first 5 years after graduating.^1^ Inspired by the annual seminar held at the recent SAAFP conference in Cape Town,^2^ this article touches on key lessons to help new family physicians enhance their leadership skills and identity.

## Navigating tension in leadership

One of the biggest challenges discussed at the seminar was the tension that may accompany leadership roles. Newly qualified family physicians often face difficult situations with fellow colleagues, patients and even themselves as they learn to cope with their growing responsibilities. Balancing clinical duties with being in a leadership position can lead to stress and uncertainty.^3^ If left unaddressed, it may impact team dynamics, patient care and mental health. To avoid this tension, the healthcare team should foster an open and transparent communication culture and embrace the key elements of ethical leadership.^4^ Expectations should be voiced, concerns should be shared, and regular feedback will build trust. This will ensure potential conflicts can be avoided before they escalate.

## Mentorship and support

Leadership can sometimes be lonely, especially in the early years after graduating and stepping into a more senior role. The seminar participants emphasised that a good professional support structure is essential for professional and personal growth. Mentorship, in particular, was mentioned as playing a role in helping newly graduated family physicians adapt to leadership responsibilities. Experienced mentors can provide guidance on navigating complex issues, managing time effectively and striking a balance between clinical and managerial duties.^5^ A mentor who can offer insight into the specific demands of the new role is invaluable.

## Embracing diversity

An important theme from the seminar was that ‘the differences make the difference’. Leading a diverse healthcare team is one of our speciality’s strengths. Teams often consist of members with different experiences, skills, and perspectives, all of which contribute to better patient outcomes and a more dynamic working environment. For leaders, recognising the value of embracing these differences is essential and has important implications for developing healthcare leadership in our context.^6^

## Leading with humility and fairness

Leadership in family medicine is inherently about service to our patients and colleagues. The seminar highlighted that authentic leadership is rooted in humility and kindness. Participants discussed how fairness manifests in how decisions are made and how team members are treated. While leaders may hold positions of power, the best leaders recognise the importance of listening to others, learning from their team and acknowledging their limitations. Humility allows leaders to create a space where collaboration flourishes and team members feel empowered to contribute.^7^ Such a values-based leadership style will help strengthen relationships and develop a suitable learning culture.^8^

## The importance of self-awareness and recognising limits

One of the most insightful topics discussed in the seminar was the importance of self-awareness. Leaders aware of their strengths, weaknesses and emotional triggers are better equipped to lead effectively. Self-awareness and emotional intelligence enable leaders to make informed decisions, delegate appropriately and seek support when necessary. Knowing one’s limits is equally important, particularly in the demanding field of family medicine in our South African context. Overextending oneself can lead to burnout, negatively affecting both the leader and the team. Self-aware leaders recognise when they need to step back, ask for help or reassess their workload.^9^

## Understanding roles and responsibilities

Finally, healthcare leadership requires a deep understanding of the roles, responsibilities and frameworks that guide our everyday practice. Newly qualified family physicians often navigate complex situations, and following the ‘I – we – it’ model may help structure an approach to dealing with these situations.^10^ Understanding the broader system within which our healthcare system operates is critical for effective leadership, whether ensuring adherence to guidelines or advocating for better resources.

## Conclusion

Leadership in family medicine is not just about position or authority. It is about fostering collaboration, addressing tensions and guiding the team with humility, kindness and fairness while understanding the roles and responsibilities of the position. The Next5 continues to offer support to newly qualified family physicians, helping them navigate the complexities of the new role in which they find themselves and providing mentorship support for those who wish to use the platform. Family physicians can become better leaders and more effective caregivers by embracing self-awareness, understanding their limits and recognising the importance of mentorship and diversity.

Register today to join the Next5 activities: https://saafp.org/next5/.
